# Retroperitoneal robot-assisted live-donor nephrectomy: A single-center study

**DOI:** 10.3389/frtra.2023.1062240

**Published:** 2023-04-26

**Authors:** Rashed Rowaiee, Mandana Gholami, Waldo Concepcion, Hemant Vedayar, Farhad Janahi

**Affiliations:** ^1^ College of Medicine, Mohammed Bin Rashid University of Medicine and Health Sciences, Dubai Healthcare City, Dubai, United Arab Emirates; ^2^ Department of General Surgery, Mediclinic City Hospital, Dubai Healthcare City, Dubai, United Arab Emirates; ^3^ Department of Urology, Mediclinic City Hospital, Dubai Healthcare City, Dubai, United Arab Emirates

**Keywords:** robot assisted live donor nephrectomy, live donor nephrectomy, kidney transplant, bowel perforation, minimally invasive technique

## Abstract

**Background:**

As the demand for kidney transplants continues to increase globally, healthcare institutions face a challenge to bridge the gap between patients waitlisted for kidney transplants and the number of donors. A major factor influencing the donor's decision is the operative risk and potential complications of the surgery. Open surgical approaches have been vastly replaced with laparoscopic donor nephrectomies as the standard of practice. However, there is a growing body of evidence pointing towards its potential superiority over laparoscopic methods. In this study, we aim to present our experience on outcomes of Robotic-Assisted Live Donor Nephrectomies (RALDN), the first series of its kind in the United Arab Emirates (UAE).

**Methods:**

We retrospectively collected data from patients who underwent RALDN at Mediclinc City Hospital. Demographic data, laboratory investigations, and operative details were collected and analyzed.

**Results:**

Seven patients underwent RALDN between 2021 and April 2022 at our facility. Four donors were male while three were female. Median length of hospital stay was 4 days. In our study, one of the patients suffered from a Clavien-Dindo grade IV complication which necessitated prolonged admission.

**Conclusion:**

We conclude that RALDN is a safe method for donor kidney procurement, carrying a low risk of morbidity and mortality. This method could potentially evolve the number of kidney donors to address the issue of high kidney transplant demand.

## Introduction

For many years, live donor nephrectomy was performed only with an open surgical approach and thereby many potential donors were reluctant to donate due to the morbidity associated with the procedure. In 1995, Ratner et al. performed the first Laparoscopic Donor Nephrectomy (LDN) which demonstrated several improvements over Open Donor Nephrectomy (ODN) such as decreased postoperative pain, length of stay (LOS), and perioperative blood loss. Due to these advantages, LDN has become the standard of care. In 2000, Horgan et al. performed for the first time a Robotic-Assisted Donor Nephrectomy (RALDN) (1). Since then, it has been adopted by several institutions worldwide and the amount of evidence has progressively increased (2). To date, RALDN represents an evolving field.

Unlike most surgical procedures, live donor nephrectomy is a unique, elective procedure, where a subject undergoes surgery for the sole benefit of another. Therefore, it is of great importance to keep the morbidity and mortality of the procedure as low as possible. Moreover, efforts should be made to procure the kidneys in optimal conditions for transplantation. The value of this study is compounded by the fast evolution of our local kidney transplantation scene.

In this study, we aim to evaluate the effectiveness of utilizing RALDN in our practice in Dubai by comparing our results from this first-ever case series to our local standard in addition to reviewing the resemblance of our data to international standards.

## Methods

We have retrospectively reviewed electronic patient records at Mediclinic City Hospital, who underwent RALDN during the year 2021 and up to April 2022. Demographic and patient-related data regarding age, gender, BMI and heigh were extracted. In addition, operation details such as operating time, estimated blood loss, renal vasculature anatomy, neohrectomy site, length of hospital stay, and complications rate were recorded. Relevant laboratory data such as pre-op and post-op creatinine were collected as well. All data were extracted into an Excel sheet for subsequent analysis. Descriptive statistics were adopted using Excel formulas.

## Technique

**Figure 1:**
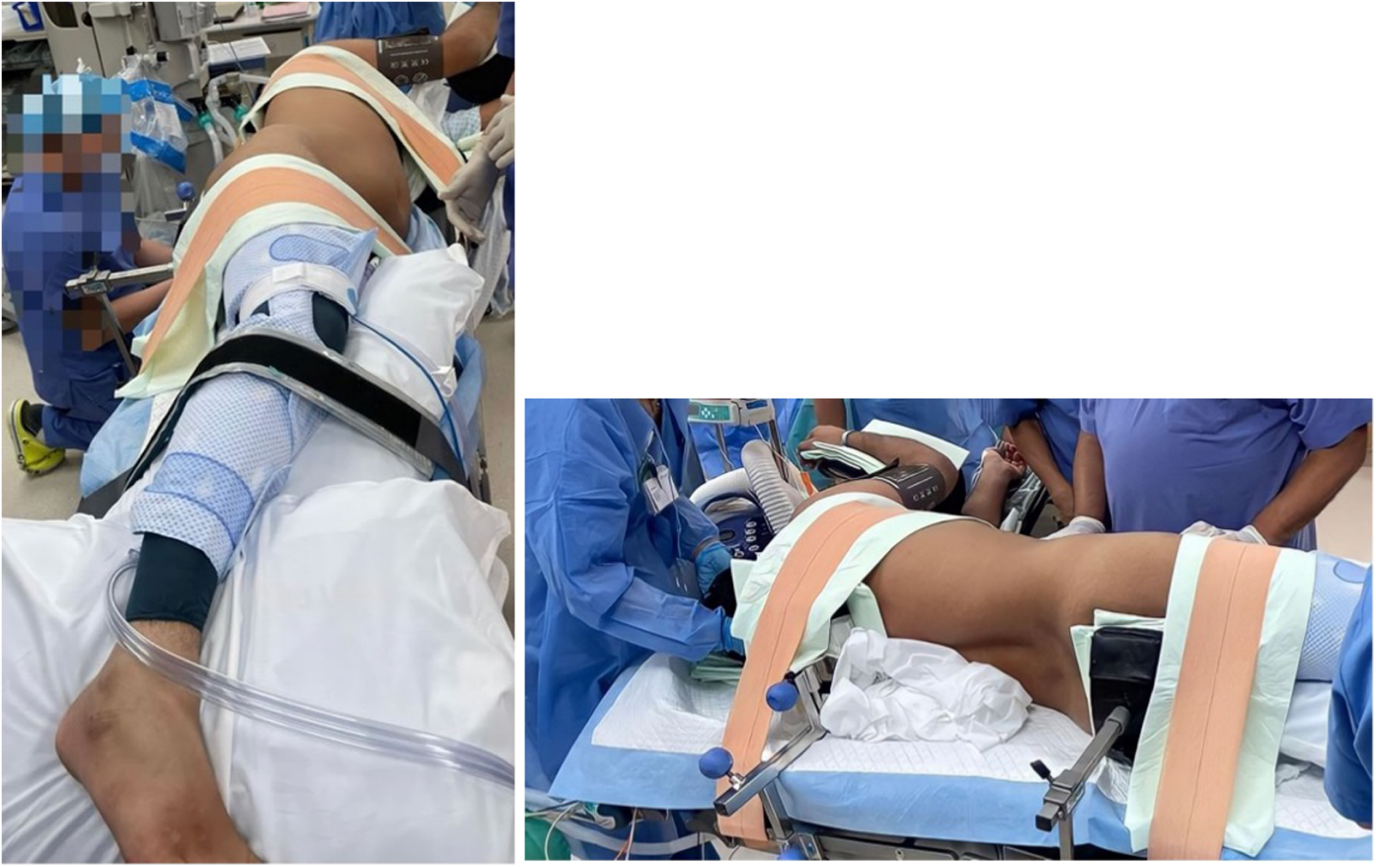
Patient positioning.

The retroperitoneal robot-assisted technique was first described by Akin et al., and has been adopted by out institution quite recently (2). After general anesthesia, the patient is positioned in the lateral decubitus position, with the operating table minimally flexed at about 30 degrees for ease of access to intended intra-abdominal anatomy. All the pressure points are carefully padded (Figure 1).

**Figure 2:**
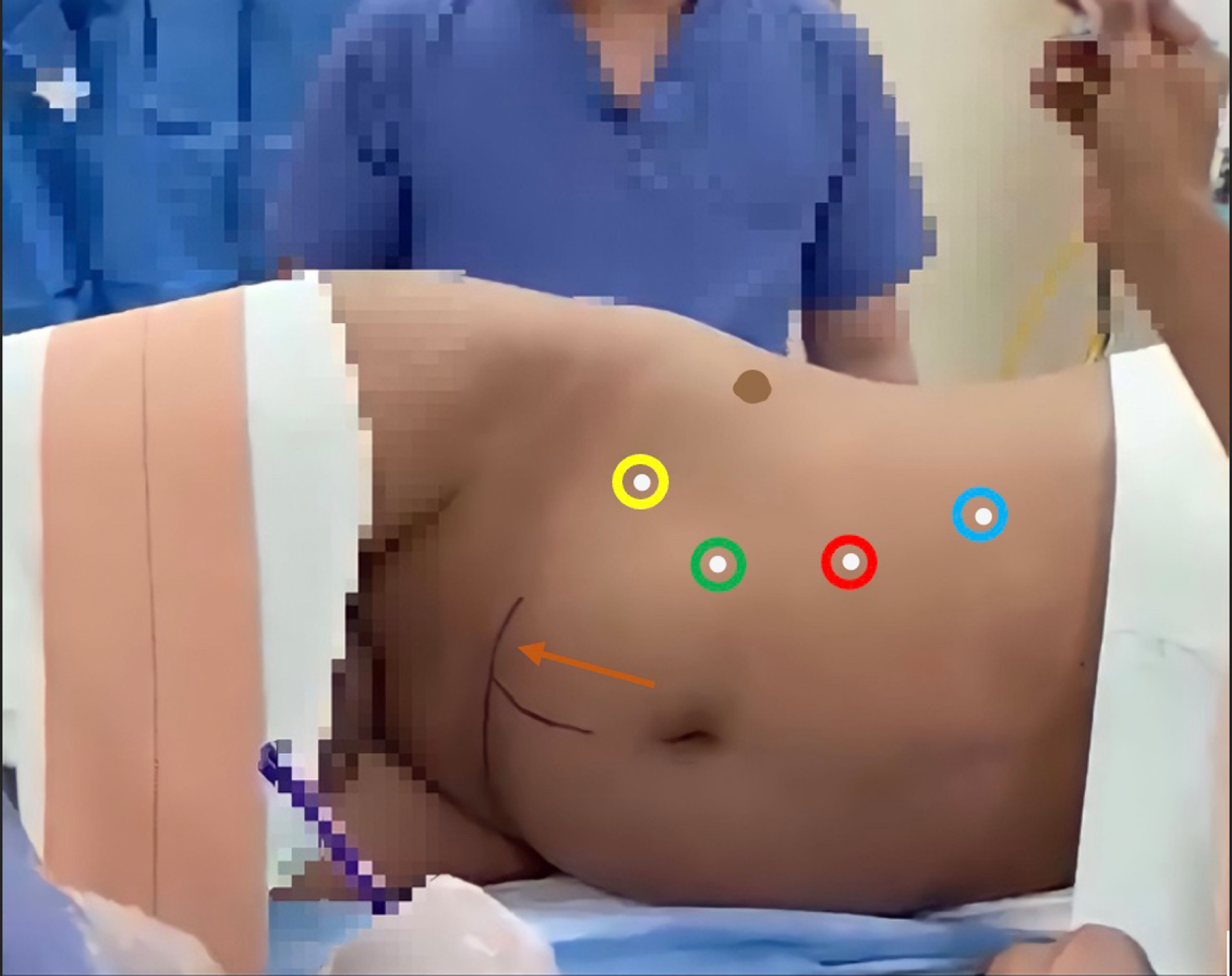
Robotic port marking. Green circle: 12 mm Camera port; Blue, red and yellow circles: operating ports, including retractors; Orange arrow: Pfannenstiel incision, and Alexis Port insertion point (Perpendicular line from the umbilicus is for surgeon's guidance only).

Our facility uses the DaVinci Xi Robot, which allows insertion of four ports. The positions of the robotic ports are first marked, which consists of a 12 mm camera port in the periumbilical region and another three 8 mm ports, in a linear fashion, from the subcostal mid-clavicular line, with about 7 cm of space between each port (Figure 2).

Initially, a 5–6 cm Pfannenstiel incision was made, which serves as the point of kidney procurement. The retroperitoneal space was then accessed by splitting the two rectus muscles. Using blunt hand dissection, the retroperitoneum is then retracted, and a space is created for the first port insertion, which is the camera port. The retroperitoneal space is then inflated. Once the peritoneal space is inflated, the remaining ports are inserted under direct visualization, perpendicular to the kidney axis. Alexis Wound Protector (Applied Medical, Rancho Santa Margarita, Calif, United States) was inserted into the Pfannenstiel incision, which has an added benefit of providing an additional 12 mm working port. The robot was then docked, with the port 1 and 3 utilised as working ports, port 2 as the camera port and port 4 for retraction.

The first step of the procedure involves dissection of the perinephric fat off of Gerota's fascia mobilizing it to the lower retroperitoneum. Next, to expose the kidney, Gerota's fascia is incised above and along the psoas muscle. The kidney is then on the lateral and inferior aspect. The ureter is then usually visualized anterior to the psoas muscle, medial to the Gerota's fascia, continuing superiorly into the hilum. The ureter was held up and dissection was carried out towards the hilum of the kidney. The left gonadal vein was identified, which was then divided by LigaSure. Next, anterior surface of the vein was dissected free until the left renal vein was identified. The suprarenal vein was identified at its insertion into the renal vein and was dissected free, and was further divided with LigaSure. Following dissection of the adrenal gland from the kidney, both branches of the renal artery is identified and dissected free from the anterior and posterior surface. Following mobilization of the ureter inferiorly, it was divided between clips and was therefore completely freed. Renal vasculature were held up and then divided with the robotic 45 stapler using white cartridges in that order. The kidney was delivered through the Pfannenstiel incision and inserted immediately in ice. The stump of renal artery and vein were carefully inspected to ensure hemostasis. The wound was then closed with Stratafux for the sheath of the Pfannenstiel incision, and 2'0 Vicryl for the subcutaneous tissue of this incision. All the remaining skin incisions were closed using 4'0 Monocryl with subcuticular incision. The kidney was perfused on the back table with HTK solution to ensure that the kidney was thoroughly cleaned of all the blood. The kidney was then packed in ice and was carried across for implantation.

## Results

A total of seven patients underwent RALDN at Mediclinic City Hospital between September 2021 and April 2022 (Table 1). Of those, three patients were female, and four were males with a mean age of 37 years old. Surgical approaches are influenced by several patient factors including their BMI and height. The mean BMI amongst our seven patients was 24.85 kg/m^2^ (Range 19.88–31.73) and the mean height of the seven patients were 167 cm (Range 157–182). In our patient cohort, six patients had their left kidney removed compared to one who had their right kidney removed due to the complicated left renal vasculature nature as described in Table 1. The average pre-operative donor creatinine was 73.6 umol/L (Range 61–118), while average post-operative creatinine was 107.9 umol/L (Range 82–136). The mean operative time was 4 h 20 min (Range 03:27–05:45). Estimated Blood Loss ranged from 5 to 50 ml with 50 ml in the first procedure conducted and a downward trend in the subsequent procedures. Due to the outlier value present in the length of the hospital stay for patient number 7, a median value, instead of mean was calculated as 4 days (Range 3–30). Since our last patient encountered a major surgical complication, he was required to be admitted to the ICU to receive appropriate level of care, prolonging his stay to up to 30 days.

**Table 1:** Patient demographic, operation and relevant laboratory data.

Patient	Age	Gender	BMI (kg/m^2^)	Height (cm)	Renal vasculature anatomy	Nephrectomy side	Pre-op creatinine (umol/l)	Post-Op creatinine (umol/l)	Operation time (hr: min)	Estimated blood loss (mL)	Length of hospital stay (days)	Complications
1	27	Male	27.17	182	Multiple arterial supply of the left kidney, with single renal vein	Right	73	118	05:45	50	3	None
					Right kidney with a single artery and vein							
2	24	Male	19.88	174	Single left renal artery and vein	Left	70	114	04:22	30	4	None
3	41	Female	31.73	157	Single left renal artery and vein	Left	62	82	03:27	5–10	3	None
4	42	Female	26.78	159	Single left renal artery and vein	Left	65	112	04:15	15–20	4	None
5	50	Female	21.42	157	Single left renal artery and vein	Left	66	85	04:26	5–10	4	None
6	52	Male	26.78	157	Two left renal artery and single vein	Left	61	108	03:49	5–10	3	None
7	23	Male	20.25	180	Single renal artery and vein	Left	118	136	04:20	5–10	30	Bowel perforation
Mean/median values (range)	37 (23–52)	-	24.85 (19.88–31.73)	167 (157–182)	-	-	73.6 (61–118)	107.9 (82–136)	4:20 (03:27–05:45)	-	Median: 4 (3–30)	

## Discussion

In this case series, we report our experience on the first ever RALDN cases within the United Arab Emirates. Although RALDN is a rapidly emerging technique for donor nephrectomies, to our knowledge, no countries within the region have reported such cases and the literature contains scarce and perhaps controversial information which requires careful evaluation.

Laparoscopic Donor Nephrectomies (LDN) has been the dominant method of donor kidney procurement since it was first introduced in 1995 (3). This has crucially impacted the number of kidney donations globally due to its superiority in safety over the open surgical method. Open surgical methods were associated with significantly longer hospital stay vs. the laparoscopic method which offers longer warm ischemia time, less intraoperative blood loss, less perioperative pain, lower graft rejection rates, and quicker rehabilitation for previously healthy donors (4–9). Laparoscopic techniques therefore remarkably revolutionized donor nephrectomies, contributing significantly to the living kidney donor pool, however, significant discrepancies still exist between patients waitlisted for kidney transplantation and the number of donors. This calls for the implementation of advanced strategies to address this high demand.

Kidney donation is an entirely altruistic act, hence the utmost care should be taken to provide the donor with the safest and most comfortable experience. In the field of urology, robotic surgeries have been well established for a variety of pathologies, offering an excellent replacement for laparoscopic techniques (10, 11). Horgan et al., were the pioneers in RALDN, performing for the first time globally in 2001, on 10 patients (1). Ever since then, various other centers adopted the method and reported on their experience in the literature (2, 12–14).

A significant advantage of utilizing robots for donor nephrectomies is decreasing the length of hospital stay. We observed a shorter mean length of stay, compared to other studies conducted- 3 days, vs. 5 days in the studies conducted by Giacomoni et al., Hubert et al., Akin et al., and Laplace et al., (2, 12, 14–16). This shorter length of stay could be attributed to the robotic technique itself which reduces peritoneal disruption, better visualization and dissection of tissue planes, and better control of minor blood vessel injuries if any encountered during the procedure (15, 17). In our investigation, we report a LOS of 7.3 days which is higher than the mean LOW for laparoscopic method, however this can be explained as an outlier which skewed the result. Other analysis excluding the outlier results in a LOS of 3.5 days. Moreover, it has been previously reported that patients who underwent minimally invasive techniques experience less pain post-operatively, and have a smoother and speedy recovery, which further factors into a shorter hospital admission period (18). This can be also confirmed by the fact that none of the donor patients had presented to the emergency department nor had been re-admitted post-procedure for pain.

We have discovered that the operative time for our study was 260 min which is higher than the standard for RALDN which stands at 175 min. This could be explained by the growing level of experience of our team in conducting this technique. We can observe the decreasing trend in operative time from patients One to Six indicating that with a rapidly developing experience with this technique, we would be able to achieve a similar operative time. This trend was also observed by Horgan et al. and many others who have adopted their institution. In our case, this rather prolonged OT time has not resulted in any complications for the patient.

When comparing RALDN against LDN, studies suggest superiority of RALDN in terms of morbidity rates, or comparably as safe as LDN with the main drawbacks of the latter being diminished vision and control, both of which are vastly rectified by the robotic approach. Donors required less post-operative pain management, had a shorter length of stay, and minimal peri-operative complications were reported (19–22).

For all of our donors, we have adopted the retroperitoneal approach to kidney procurement. Although retroperitoneal partial nephrectomies were established in the literature, Akin et al., were the first to introduce the retroperitoneal (RP) approach for live donor nephrectomies (2). Several studies have evaluated surgical outcomes of transperitoneal (TP) approach compared to the retroperitoneal. One of the most significant advantages of which is consistent amongst almost all studies is the shorter operative time associated with RP approach (23–25). This is best explained by the RP approach eliminating the need to mobilize the bowel or the kidney during the surgery (23, 24). Eraky et al., were the first to also confirm the shorter time to access the renal hilum, which could serve as another reason for the shorter operative times observed by the RP approach (26). Another added benefit of the RP approach is the decreased blood loss and subsequently the need for blood transfusion (23, 24). The ease of access to the renal hilum due to the lesser need for dissection, could be a potential reason for this observed outcome (23, 27). Additionally, the RP approach could contain any hemorrhage or urine leak, which could be another reason for less blood loss observed (2). Furthermore, RP approach can be safely used for patients with previous adhesions or scars from past surgeries, which is a limitation of the TP approach (23). With regards to complications, the incidences of both major and minor complications seemed comparable between the two approaches (23–25).

Although no randomized control studies (RCT) exist to confidently determine the superiority of either method, Zhou et al. have performed an updated meta-analysis on the available literature (28). The results of the meta-analysis exhibit an agreement with the previous studies in terms of superiority of RP on operation duration, and less blood loss. Moreover, they have observed a shorter postoperative hospital stay. This has also been explained by the lack of need of bowel and mobilization and peritoneal manipulation, which subsequently translates into protection of the abdominal organs from hematoma and urine leaks, as well as postoperative ileus, enhancing the postoperative recovery period (28). However, similar to aforementioned studies, neither approach exhibited superiority regarding postoperative complications (28).

It is worth noting that all the aforementioned studies are conducted for robotic partial nephrectomies for renal tumours. To the knowledge of authors, no studies to this date exist comparing TP and RP approaches in robotic living donor nephrectomies, which could serve as a potential area of further research.

As is the case for all surgical procedures, there are still complications and pitfalls of RALDN especially given the relative novelty of this procedure. The most commonly reported intra-operative complication was bleeding, most frequently of the renal arteries (17, 19, 29). To our knowledge, no bowel perforation cases have been reported in the literature following RALDN. However, bowel perforation has been reported across several studies, following laparoscopic live donor nephrectomies (30–32). Most commonly, these perforations and bowel injuries were observed to be a result of instrumental lesion or injuries sustained by the stapler (8, 32). In the case of this patient, developing a Clavien-Dindo grade IVa complication given the procedure and approach and technique is almost unheard of. A key strength of the retroperitoneal approach is the lack of need to mobilize the colon during dissection which limits the chances of bowel injury, from our patient we can certainly conclude that the chances are not zero. It is postulated that even though the colon was not directly mobilized by the robotic arms, the heat generated by the electrocautery system in the robotic hands may have caused the colonic perforation in the case of our patient.

Thus, we should critically highlight this scenario to draw on the lessons from it and spread awareness to the wider medical community.

Surgeon's experience plays a crucial role in preventing untoward donor-related complications, where most of the complications are believed to occur at the beginning of the learning curve. As observed in previous studies, with increased number of RALDNs carried out, the number of major complications, namely bleeding and the need to convert to open surgery has been significantly reduced over time (29, 33).

Additionally, RALDN has been postulated to have significant strength when it comes to complex cases such as those with an additional renal artery or lumbar vein. Pre-operative angiogram could be the decision-maker for the technique of choice for pre-operative planning of donor nephrectomies in which the robotic approach presents better safety to the donor (Tae young shin). Gorodner et al., compared the safety of the procedure in two groups of patients, with and without renal vascular anomalies. In their study, they concluded that there was no significant difference regarding blood loss, LOS, or conversion to open operation in the group of vascular anomaly vs. the control.

Moreover, laparoscopic surgeries may potentially carry a risk of intra-operative blood loss, however, as reported in our experience, there was minimal difference in pre- and post-op hemoglobin levels (9, 22). This was consistent in other studies as well, further affirming the low risk of operative complications with RALDN.

In our investigation, we have encountered several limitations which should be addressed. Firstly, the authors acknowledge the small sample size with the single center scope. However, in the Middle East, the donor pools are relatively less compared to the West, and additionally, robotic methods of kidney retrieval is still a novel approach in Dubai, as well as within the region and as mentioned previously, to our knowledge, this study is the first of its kind within the region. Additionally, our sample did not contain any patients with comorbidities that could complicate the procedure such as obesity, smoking, or diabetes mellitus. We would encourage future studies to take into account those patients who have impaired wound healing ability and conduct studies with multivariate analysis to evaluate these populations, which later could increase the external validity of the study.

## Conclusion

This is the first-ever study reporting on RALDN in the UAE and within the region. Given our results amongst the first 6 patients who underwent RALDN, we conclude that RALDN is a safe, effective way of donor kidney retrieval carrying a low risk of morbidity. In times of high demand for donor kidneys, RALDN implementation should be highly encouraged across the region, in an attempt to increase the body of kidney donations. We hope that the data generated from this experience will be beneficial to academic medical institutions globally and pose a possible strategy for constantly rethinking resource management and optimizing patient care.

## Data Availability

The raw data supporting the conclusions of this article will be made available by the authors, without undue reservation.
